# Risk Perception for Developing Cardiometabolic Disease Among Women Diagnosed with Preeclampsia and Gestational Diabetes Mellitus

**DOI:** 10.1089/whr.2024.0090

**Published:** 2024-11-06

**Authors:** Constance Busvine, Sarah Halmer, Alena Rosenauer, Susanne Schubert, Sascha Klee, Alexander Lösch, Martin Wiesholzer, Barbara Wichert-Schmitt, Birgit Pfaller

**Affiliations:** ^1^ Karl Landsteiner University of Health Sciences, Krems, Austria.; ^2^ Department of Internal Medicine 1, University Hospital St. Pölten, St. Pölten, Austria.; ^3^ Department of Obstetrics and Gynecology, University Hospital St. Pölten, St. Pölten, Austria.; ^4^ Division of Biostatistics and Data Science, Karl Landsteiner University of Health Sciences, Krems, Austria.; ^5^ Medical Faculty, Department of Cardiology and Medical Intensive Care, Kepler University Hospital, Johannes Kepler University, Linz, Austria.

**Keywords:** preeclampsia, gestational diabetes mellitus, risk perception, cardiometabolic disease

## Abstract

**Background:**

Adverse pregnancy outcomes (APO), including preeclampsia (PE) and gestational diabetes mellitus (GDM), increase the future risk of developing cardiometabolic disease (CMD), such as cardiovascular disease and type 2 diabetes mellitus (T2DM). CVD is the leading cause of death among women. Despite the well-established relationship between APO and CMD, women’s awareness is limited. We aimed to assess and compare risk perception for future CMD among women with PE and GDM.

**Methods:**

Women diagnosed with PE and GDM between 2015 and 2020 at the University Hospital St. Pölten were identified. Telephone interviews were conducted to assess women’s risk perception of future CMD.

**Results:**

Of the 161 women included in the study, approximately half had a high risk perception of future CMD. Women with PE (*n* = 46) were less aware of their long-term risks than those with GDM (*n* = 115), and 43.5% were unaware of any association between APO and CMD. Risk perception increased among all the participants when they considered their future CMD risk without lifestyle changes. Women with high risk perceptions were three times more likely to plan on modifying their lifestyle behaviors in the future to mitigate their CMD risk.

**Conclusion:**

This study demonstrated a lack of risk perception for future CMD among women with APO, underscoring the need for improved patient education during and after pregnancy. To increase risk perception, it is crucial to educate women about the long-term risks associated with APO, emphasize the severity of CMD, and promote lifestyle interventions.

## Introduction

Cardiometabolic disease (CMD), including cardiovascular disease (CVD) and type 2 diabetes mellitus (T2DM), is an illness that affects an individual’s cardiovascular system and metabolic health.^1^ CVD is the leading cause of death in women, accounting for 47% of all female deaths in Europe.^2^ However, significant gender disparities exist in the diagnosis and treatment of CVD; women are less aware of their cardiovascular risks, underrepresented in clinical trials, often present with “atypical” symptoms, and are thus underdiagnosed and less likely to receive adequate guideline-based treatment.^3–5^

CMD shares common risk factors, including obesity, smoking, dyslipidemia, hyperglycemia, and hypertension.^6^ In addition, women experience unique risk periods such as pregnancy.^7^ During pregnancy, extensive anatomical and physiological changes occur to support fetal development.^8^ Most pregnant women tolerate these changes; however, approximately 20% develop adverse pregnancy outcomes (APO).^9^ APOs comprise events such as hypertensive disorders of pregnancy, gestational diabetes mellitus (GDM), and preterm delivery.^10^

Preeclampsia (PE) is a hypertensive disorder of pregnancy affecting 2%–8% of pregnant women worldwide.^11^ Women with PE have a four-fold increased risk for hypertension and heart failure, as well as a two-fold increased risk for T2DM, coronary heart disease, and stroke.^11–14^

GDM affects 14% of pregnancies worldwide and is associated with an almost 10-fold increased risk of developing T2DM, with about half of the affected women developing GDM in a subsequent pregnancy.^15–18^ Furthermore, GDM doubles the risk of future CVD events.^19^

Despite the well-established link between APO and CMD, health care providers (HCP) and women alike have limited awareness of the increased future risk of developing CMD following APO.^20^ A study published in the United States found that among 182 women with APO, only 48% reported that they had a higher than average risk of developing CVD.^21^ A cross-sectional analysis of 79 women with PE and GDM found that only 36.4% and 45.7%, respectively, had a high risk perception of future chronic disease.^22^ In a Canadian study of 902 women with GDM, close to half perceived themselves to be at low risk for developing T2DM within the next 10 years.^23^ A recent Austrian study of women with PE and GDM found that only one-third received counseling from an HCP about their future CVD risk. Women with PE were less likely to receive postpartum care or lifestyle recommendations compared with those with GDM, and only one-fifth of all participants were informed about the need for long-term follow-up.^24^

Risk perception is “an individual’s perceived susceptibility to a [health] threat.”^25^ Studies have shown that a high risk perception for future diseases is associated with an increased willingness to adopt health-protective behaviors, including lifestyle changes and regular health assessments.^22,25–28^ Many factors influence risk perception, and this study will analyze four behavioral modifiers of risk perception as follows: (1) personal control or the perception that an individual can influence the development of CMD through their actions; (2) optimistic bias, defined as an individual’s belief that, compared with others, they are less likely to develop CMD; (3) knowledge of CMD risk factors; and (4) the belief in the benefits of preventative measures.^29–32^

This study aimed to assess women’s awareness of their CMD risk following a diagnosis of PE or GDM and compare differences in risk perception between the two groups.

## Methods

### Study design

Women with PE and GDM who delivered at the University Hospital St. Pölten, Austria, from January 1, 2015, to October 31, 2020, were identified via the hospital information system. In total, 750 women received an invitation letter to participate in the telephone interview. Women were included if they were German-speaking, 18 years or older at the time of delivery, and if electronic records confirmed a diagnosis of PE or GDM in their most recent pregnancy (B.P.). Due to the study period (2015–2020), PE was defined according to the 2013 American College of Obstetricians and Gynecologists Task Force on Hypertension in Pregnancy.^33^

Of the 750 women identified, 589 were excluded because they did not meet the inclusion criteria, had no valid contact information, or declined to participate. The final cohort comprised 161 women.

From June to October 2021, trained interviewers (A.R. and C.B.) conducted structured telephone interviews after obtaining oral consent.

A custom survey was designed, as no validated German survey instrument exists to assess women’s risk perception and knowledge of CMD. The participants were asked about their demographics, medical and obstetric history, family history, physical measurements, postpartum care counseling, risk perception for future CMD, and counseling on cardioprotective strategies. Data pertaining to women’s medical and obstetric history were supplemented using their electronic medical records.

For women with GDM, risk perception for T2DM was examined using the “Risk Perception Survey for Developing Diabetes.”^29,30^ For women with PE, the survey was adapted to include risk perception for cardiovascular events.^34^ Risk perception for CMD was assessed by asking women, “What do you think your risk or chance is for developing T2DM/hypertension or CVD over the next 10 years?”. The answer options included the following: “a high chance,” “a moderate chance,” “a slight chance,” “almost no chance,” and “I do not know.” Another question examined women’s plans regarding lifestyle changes by asking, “If you don’t change your lifestyle behaviors, such as diet or exercise, what is your risk or chance of developing T2DM/hypertension or CVD over the next ten years?”. Women were also asked about past lifestyle behavior changes and their plans to change their future lifestyles.

Further questions assessed the four behavioral modifiers of risk perception.^29,30,32^ Participants were able to answer using a 4-point Likert scale, with the following responses: “strongly agree,” “agree,” “disagree,” “strongly disagree,” and “I do not know.”

Women’s future risk awareness of CMD was determined by asking participants to state the long-term risks following their pregnancy complications. Possible responses included T2DM, hypertension, stroke, coronary heart disease, myocardial infarction, or no long-term risk.

### Institutional review board statement

The study was conducted following the Declaration of Helsinki and approved by the Commission for Scientific Integrity and Ethics of the Karl Landsteiner University of Health Sciences (1099/2020, April 18, 2021).

### Statistical analysis

SPSS Version 29 (Windows, IBM Corp. Armonk, New York) was used for the data analysis. Data were presented as means ± standard deviations and categorical variables as proportions. Chi-square, Student’s *t*-test, or Fisher’s exact test was used, when appropriate, to compare women with PE with those with GDM. A two-sided *p*-value <0.05 was considered significant.

Risk perception of CMD was classified into two groups. Women were classified as having “low risk perception” if they perceived themselves to have a “slight” or “almost no chance” of developing CMD. Women who perceived themselves to have a “moderate” or “high chance” of developing CMD were classified as having “high risk perception.” Women were excluded from statistical analysis if they responded, “I do not know.” Regarding the behavioral modifiers of risk perception, the answers “agree” and “strongly agree” were combined, as were the answers “disagree” and “strongly disagree.” This resulted in two categories, namely “agree” and “disagree.” Regarding the awareness of long-term risks, a Bonferroni correction was applied such that a *p*-value <0.007 denoted a significant difference between the two groups.

## Results

### Baseline characteristics

Of the 161 women included in our study, 46 (29%) were diagnosed with PE, and 115 (71%) had GDM. Women with GDM were more likely to participate in regular physical activities than those with PE. Overall, prepregnancy body mass index (BMI) was 26.5 kg/m^2^, and more than one-quarter of all participants were obese (BMI >30 kg/m^2^). The percentage of women with obesity did not differ significantly between the two groups. Maternal and gestational ages at delivery were significantly lower in women with PE than in those with GDM. Among women with GDM, approximately one-third were older than 35 years at the time of delivery compared with six women with PE (*p* = 0.022). Almost all the women with GDM (96.5%) were diagnosed before 28 weeks of gestation. Twenty-eight women (60.9%) with PE were diagnosed with early-onset PE (before 34 weeks of gestation), and almost one-fifth had chronic hypertension and thus developed superimposed PE.^35^ Close to half the participants had a cesarean delivery; however, women with PE were twice as likely to have one than women with GDM (78.3% vs. 36.5%, *p* < 0.001). Among the women with GDM, 16 had a history of GDM. Approximately half of all participants had a family history of chronic hypertension, 67.4% of women with PE, and 46.1% of those with GDM (*p* = 0.02). Approximately 50% of women reported a family history of type 1 diabetes mellitus or T2DM, 56.5% of women with GDM, and 37.0% with PE (*p* = 0.03). Significantly more women with PE had a prolonged hospital stay due to pregnancy complications than women with GDM, and three women with PE were admitted to the intensive care unit (ICU) following delivery. None of the women was admitted to the ICU during pregnancy (Table 1).

**Table 1.** Baseline Characteristics of Women Diagnosed with Gestational Diabetes Mellitus or Preeclampsia^24^

Variable	Total (*n* = 161)	GDM (*n* = 115)	PE (*n* = 46)	*p*-value
Maternal age at survey, mean ± SD	35.2 (±5.3)	36.0 (±5.3)	33.4 (±5.0)	0.006
Ethnicity, *n* (%)				0.63
Caucasian	154 (95.7)	108 (93.9)	46 (100.0)	
Southeast Asian	3 (1.9)	3 (2.6)	0 (0.0)	
African—Black	1 (0.6)	1 (0.9)	0 (0.0)	
Other	3 (1.9)	3 (2.6)	0 (0.0)	
Married, *n* (%)	107 (66.5)	78 (67.8)	29 (63.0)	0.80
Regular physical activity, *n* (%)	103 (64.0)	78 (67.8)	25 (54.3)	0.12
Pregnancy and delivery				
Prepregnancy BMI >30, *n* (%) (*n* = 160)	42 (26.1)	31 (27.0)	11 (24.0)	0.7
Maternal age >35, *n* (%)	41 (25.5)	35 (30.4)	6 (13.0)	0.022
Parity, *n* (%)				
1	43 (26.7)	27 (23.5)	16 (34.8)	0.68
2	60 (37.3)	42 (36.5)	18 (39.1)	
>2	58 (36.0)	46 (40.0)	12 (26.1)	
Gestational age at delivery, weeks, mean ± SD (*n* = 160)	37.8 (±3.5)	38.8 (±2.8)	35.4 (±3.8)	<0.001
Cesarean delivery, *n* (%)	78 (48.4)	42 (36.5)	36 (78.3)	<0.001
Maternal medical history				
GDM diagnosis at, *n* (%)				
<22 weeks	24 (14.9)	24 (20.9)	—	—
<28 weeks	87 (54.0)	87 (75.7)	—	—
>29 weeks	4 (2.5)	4 (3.5)	—	—
PE diagnosis at, *n* (%)				
<34 weeks	28 (17.4)	—	28 (60.9)	—
>34 weeks	18 (11.2)	—	18 (39.1)	—
Chronic hypertension, *n* (%)	11 (6.8)	3 (2.6)	8 (17.4)	0.002
Prior PE, *n* (%)	6 (3.7)	4 (3.5)	2 (4.3)	1.0
Prior GDM, *n* (%)	16 (9.9)	16 (13.9)	0 (0.0)	0.006
Prolonged hospital stay due to pregnancy complication, *n* (%)	19 (11.8)	2 (1.7)	17 (37.0)	<0.001
ICU admission, *n* (%):				
During pregnancy	0 (0.0)	0 (0.0)	0 (0.0)	—
Following pregnancy	3 (1.9)	0 (0.0)	3 (6.5)	0.006
Hospital admission in the first 6 months postpartum, *n* (%)	7 (4.3)	5 (4.3)	2 (4.3)	1.0
Family history				
Chronic hypertension, *n* (%)	84 (52.2)	53 (46.1)	31 (67.4)	0.02
PE, *n* (%)	11 (6.8)	6 (5.2)	5 (10.9)	0.30
T1DM or T2DM, *n* (%)	82 (50.9)	65 (56.5)	17 (37.0)	0.03

Values are represented as mean ± SD or *n* (%).

BMI, body mass index; ICU, intensive care unit; GDM, gestational diabetes mellitus; PE, preeclampsia; SD, standard deviation; T1DM, type 1 diabetes mellitus; T2DM, type 2 diabetes mellitus.

### Risk awareness and knowledge of long-term risks of developing CMD

Overall, close to 50% of all women were aware that CVD (stroke, coronary heart disease, and myocardial infarction) was a future risk following their pregnancy complications.

**FIG. 1.**
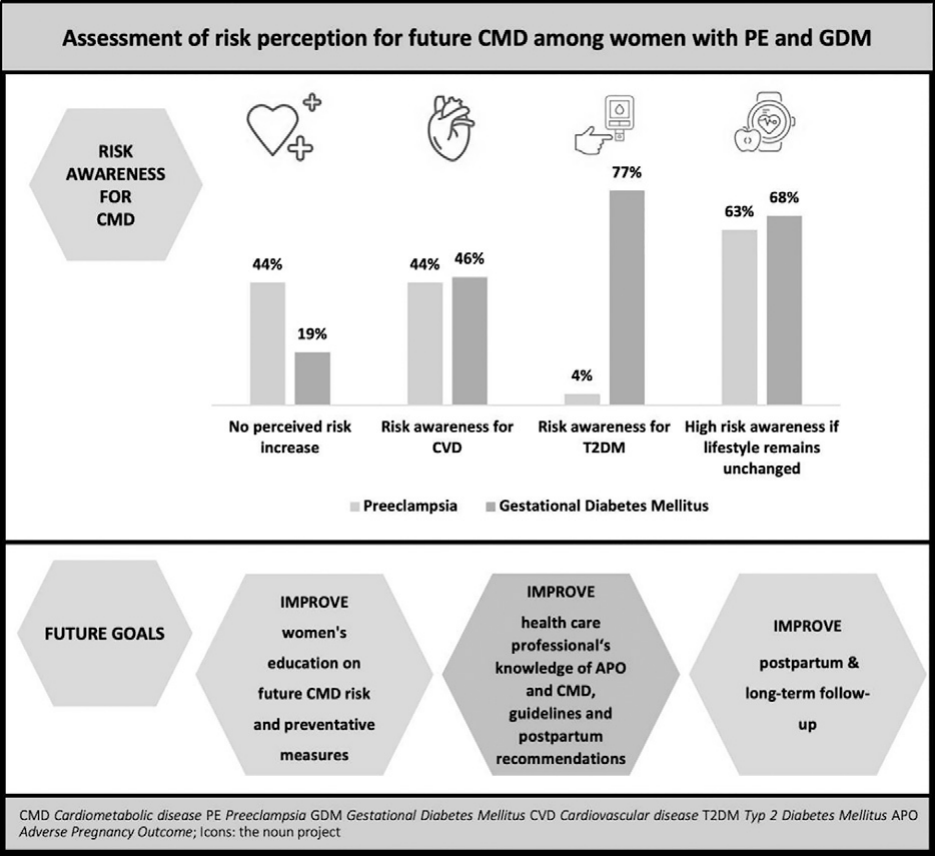
Assessment of risk perception for future cardiometabolic disease (CMD) among women with preeclampsia (PE) and gestational diabetes mellitus (GDM). Risks associated with PE and GDM, perception of these risks, and management considerations for women with PE and GDM (Central Illustration).

One-half of the women with PE were aware of their increased risk of being diagnosed with hypertension later in life; however, only two women with PE could state T2DM as a possible future risk. In contrast, more than three-fourths of the participants with GDM were aware of their long-term risk of developing T2DM (77.4% vs. 4.3%, *p* < 0.001). Approximately 30% of all women were aware of their future risk of developing a stroke, 28.7% of women with GDM, and 26.1% with PE (*p* = 0.74). More women with GDM stated that coronary heart disease was a long-term risk following their pregnancy complication compared with women with PE, although this difference was not significant (43.5% vs. 37.0%, *p* = 0.45). In total, approximately one-fourth of all women stated that they had no long-term risks following their pregnancy complications (26.1%). In women diagnosed with PE, 40% were unaware of any long-term risks following PE, which was significantly higher than in women with GDM (43.5% vs. 19.1%, *p* = 0.001) (Figure 1).

### Association between risk perception for CMD and pregnancy complication

Overall, risk perception for developing CMD was low. Less than half of all women had a high perceived risk of developing CMD in the next 10 years, and there was no significant difference in risk perception between women with GDM and those with PE (45.6% vs. 43.5%, *p* = 0.86). However, risk perception for CMD increased when women were asked to estimate their risk of developing CMD if their lifestyles did not change. Approximately two-thirds of all women had a high perceived risk for future chronic disease, 67.5% with GDM and 63.0% with PE (*p* = 0.71). Approximately half of the women had already made lifestyle changes at the time of the survey (49.6% vs. 47.8%, *p* = 0.86), and almost 70% were planning to change their lifestyle to reduce their risk of developing CMD in the future (67.8% vs. 69.6%, *p* = 0.85) (Table 2).

**Table 2.** Risk Perception for Type 2 Diabetes and Cardiovascular Disease After Diagnosis of Gestational Diabetes Mellitus and Preeclampsia, Respectively

Question	GDM (*n* = 115)	PE (*n* = 46)	*p*-value
What do you think your risk or chance is for developing T2DM/hypertension or CVD^a^ over the next 10 years? *n* (%) (*n* = 160)			0.86
High perceived risk	52 (45.6)	20 (43.5)	
Low perceived risk	62 (54.4)	26 (56.5)	
If you don’t change your lifestyle behaviors, such as diet or exercise, what is your risk or chance of developing T2DM/hypertension or CVD^a^ over the next 10 years? *n* (%) (*n* = 160)			0.71
High perceived risk	77 (67.5)	29 (63.0)	
Low perceived risk	37 (32.5)	17 (37.0)	
Have you recently made changes in any lifestyle behaviors that you believe will lower your chances of developing T2DM/hypertension or CVD^a^? (yes), *n* (%)	57 (49.6)	22 (47.8)	0.86
Are you planning to make changes in any lifestyle behaviors in the near future that you believe will lower your chances of developing T2DM/hypertension or CVD^a^? (yes), *n* (%)	78 (67.8)	32 (69.6)	0.85

Values are represented as *n* (%).

^a^ T2DM pertains to women with GDM while hypertension or CVD pertains to those with PE.

CVD, cardiovascular disease.

Compared with women with a low risk perception of CMD, women with a high risk perception were almost three times more likely to plan to modify their lifestyles in the future to decrease their risk of developing CMD (odds ratio 2.752, 95% confidence interval [1.275–5.941], *p* = 0.010).

### Behavioral modifiers of risk perception

Regarding personal control, more than 90% of all women understood that people who try to control the risks of developing CMD are less likely to develop CMD (92.1% vs. 93.0%, *p* = 1.0). Almost all women were aware that their personal efforts could control their risks of developing CMD in the long term (97.4% vs. 97.8%, *p* = 1.0), and a significant majority stated that they could take preventive measures to reduce their future CMD risk (88.6% vs. 86.7%, *p* = 0.79). Three-quarters of women with GDM stated that they had control over the risks to their health, compared with 63% of women with PE. Regarding optimistic bias, close to 90% of all women were aware that they were more likely to develop CMD in the future than other people of the same age and sex (87.6% vs. 84.1%, *p* = 0.60). Despite this, about 50% of women perceived themselves to be at a lower risk for a serious disease than similarly aged women (49.1% vs. 47.7%, *p* = 1.0). Overall, knowledge of CMD risk factors and belief in the benefits of preventative measures were high. Most women recognized that having a family member with CMD, being 65 years or older, and having APO increases the risk of developing CMD in the future. Almost all women were aware of the benefits of regular exercise, a nutritious diet, and maintaining their body weight in decreasing the risk of CMD. Approximately 40% of all women stated that exercise and following a diet required a lot of effort (39.1% vs. 43.5%, *p* = 0.72); however, about 90% of women reported that the benefits of these measures outweigh the effort required to do them (91.2% vs. 89.1%, *p* = 0.77) (Table 3).

**Table 3.** Behavioral Modifiers of Risk Perception in Women Diagnosed with Gestational Diabetes Mellitus or Preeclampsia

	GDM (*n* = 115)	PE (*n* = 46)	*p*-value
Personal control			
I feel I have little control over risks to my health (disagree^a^), *n* (%) (*n* = 160)	87 (76.3)	29 (63.0)	0.12
If I am going to develop T2DM/CVD^c^, there is not much I can do about it (disagree), *n* (%) (*n* = 159)	101 (88.6)	39 (86.7)	0.79
I think that my personal efforts will help control my risks of developing T2DM/high blood pressure or CVD^c^ in the long term (agree^b^), *n* (%) (*n* = 160)	112 (97.4)	44 (97.8)	1.0
People who make a good effort to control the risks of developing T2DM/high blood pressure or CVD are much less likely to develop T2DM/high blood pressure or CVD^c^ (agree), *n* (%) (*n* = 157)	105 (92.1)	40 (93.0)	1.0
Optimistic bias			
Compared with other people of my same age and sex, I am less likely than they are to develop T2DM/high blood pressure or CVD^c^ (disagree^a^), *n* (%) (*n* = 149)	92 (87.6)	37 (84.1)	0.60
Compared with other people of my same age and sex, I am less likely than they are to develop a serious disease (agree^b^), *n* (%) (*n* = 150)	52 (49.1)	21 (47.7)	1.0
Knowledge of risk factors for T2DM/CVD			
Having a blood relative with T2DM/high blood pressure or CVD increases the risk of developing T2DM/CVD^b^ (agree^a^), *n* (%) (*n* = 149)	98 (90.7)	36 (87.8)	0.76
Being 65 or older increases the risk of developing T2DM/CVD^b^ (agree), *n* (%) (*n* = 139)	90 (90.0)	32 (82.1)	0.25
Exercising regularly decreases the risk of developing T2DM/CVD^b^ (agree), *n* (%) (*n* = 157)	106 (95.5)	44 (95.7)	1.0
Eating a healthy diet decreases the risk of developing T2DM/CVD^b^ (agree), *n* (%) (*n* = 158)	108 (96.4)	44 (95.7)	1.0
Controlling weight gain decreases the risk of developing T2DM/CVD^b^ (agree), *n* (%) (*n* = 155)	106 (96.4)	42 (93.3)	0.67
Having had GDM/hypertensive disorders of pregnancy^c^ during pregnancy increases the risk of developing T2DM/CVD^b^ (agree), *n* (%) (*n* = 140)	92 (91.1)	31 (79.5)	0.08
Belief in the benefits of preventive measures			
Doing regular exercise and following a diet take a lot of effort (disagree^a^), *n* (%)	45 (39.1)	20 (43.5)	0.72
Regular exercise and diet may prevent T2DM/high blood pressure or CVD^c^ from developing (agree^b^), *n* (%)	115 (100)	46 (100)	1.0
Benefits of following a diet and exercise program outweigh the effort to do it (agree), *n* (%) (*n* = 160)	104 (91.2)	41 (89.1)	0.77

Values are represented as *n* (%).

^a^ Disagree indicated as 3 or 4 on the Likert scale.

^b^ Agree indicated as 1 or 2 on the Likert scale.

^c^ T2DM pertains to women with GDM, while high blood pressure or CVD pertains to those with PE.

## Discussion

Women diagnosed with APO, such as PE and GDM, have a significantly increased risk of developing CMD, including CVD and T2DM. This study assessed risk perception for future CMD, awareness of long-term risks for CMD, and behavioral modifiers of risk perception among women diagnosed with PE and GDM. Overall, risk perception for future CMD was low; among women with PE, it was slightly lower compared with women with GDM.

### Risk perception for CMD is low among women with PE and GDM

Despite the high level of awareness of the risk factors for CMD and the benefits of preventative measures among women with APO, risk perception for CMD was low. Only 45.6% of women with GDM and 43.5% with PE had a high risk perception for developing CMD in the next 10 years. Several studies reported similar results: among women with APO, the percentage of women with a high perceived risk for CMD ranged from 36.4% to 57.0%.^21–23,32,36^ An explanation for the low risk perception in our study may be that women were asked to assess their risk of developing CMD within the next 10 years rather than during their lifetime. This may have led to low risk perception, as CMD is typically associated with older age. However, in the decade following pregnancy, one-third of women with PE develop hypertension and approximately 20% of women with GDM are diagnosed with T2DM.^37–39^ This highlights the need for improved educational interventions to inform women not only of their heightened lifetime risk for developing CMD, but also the potential for early onset of CMD.

### Differences in risk perception for CMD: lower among women with PE

Overall risk perception was low, but women with PE had a slightly lower risk perception of CMD than women with GDM. Only 50% and 43.5% of women with PE identified hypertension and CVD, respectively, as long-term risks following their pregnancy complications. Similarly, a study published in the United States^34^ found that among women with PE with severe features, 64.8% correctly identified hypertension as a future risk; however, less than one-third of women with PE, regardless of severity, associated PE with an increased risk for myocardial infarction or stroke. In addition, our study found that 43.5% of women with PE assumed that they had no long-term risks. Roth et al.^20^ found that women with hypertensive disorders of pregnancy have limited knowledge about the correlation between these conditions and future CVD. These findings are concerning as women with PE have a two- to four-fold increased risk of developing hypertension and CVD, highlighting the need for improved patient education during and after pregnancy among women with hypertensive disorders of pregnancy.^11,12^

Risk perception for CMD may be lower in women with PE for many reasons. First, there is a standard of care for GDM screening in Austria. This involves screening *all* pregnant women using the “one-step” oral glucose tolerance test between 24 and 28 weeks of gestation.^40^ Following the diagnosis of GDM, women are followed up according to well-established standards in diabetes clinics. A similar formal structure does not exist for women with PE. Thus, pregnant women are more likely to receive information about GDM, its management, and its long-term consequences from an HCP compared with PE. Second, GDM is more common than PE, leading to a higher level of awareness of the condition within the population and increased public health efforts. For instance, the Austrian Diabetes Association created a brochure about GDM, which includes information about GDM, its risk factors, screening, treatment, and long-term consequences.^41^ In Austria, no medical organization has created an information brochure regarding PE. Third, the diagnosis and management of GDM are more intrusive than those of PE. Women with GDM undergo oral glucose tolerance tests and daily self-blood glucose monitoring, and up to 30% require subcutaneous insulin injections.^42^ In comparison, PE is largely diagnosed using relatively simple methods, such as urinalysis and blood pressure measurements, and treatments (*i.e.*, antihypertensives) are usually administered orally.^43^ Although few studies exist relating risk perception to intrusive procedures, a cross-sectional study examining risk perception for T2DM among 217 women with prior GDM found that subcutaneous insulin injections may increase perceptions of disease severity, leading to increased risk perception for T2DM. The study also found that the additional teaching and monitoring needed during the initiation and stabilization of insulin therapy might further contribute to the increased risk perception for T2DM.^32^ However, regardless of insulin therapy, women with GDM receive more advice and guidance from an HCP than women with PE. To establish nutritional therapy, women with GDM are often sent to specialized diabetes clinics or dieticians.^42^ Moreover, to ensure that women with GDM achieve euglycemia, the Austrian Diabetes Association^40^ recommends follow-up appointments at least every 3 weeks. At these appointments, the HCP can teach women about GDM, its future risks, and provide counseling on preventative measures. This, however, is not the case for women with PE. Therefore, women with PE have fewer opportunities to interact with HCP and learn about PE and its consequences during their pregnancies. Interestingly, our study found that 37.0% of women with PE had a prolonged hospital stay (longer than 5 days) due to their pregnancy complication, compared with only two women with GDM. A prolonged hospital stay would provide the perfect opportunity to provide women with PE information about the pregnancy complication and its long-term risks. However, a qualitative study found that women with PE often receive inadequate and inconsistent information regarding PE and its consequences. Many women are not informed that PE can progress to eclampsia, continue postpartum, develop in a subsequent pregnancy, or increase their risk of developing CVD in the future. In addition, women with PE felt unable to “take in” information because they were more concerned about the health of their babies than their own.^44^

All these reasons underscore the need for enhanced antepartum counseling, but more importantly, highlight the urgent need for improved postpartum counseling for women with PE to increase their risk perception for CMD.

### Optimistic bias and awareness of CMD severity

Women with APO were not directly asked about their lifetime risk perception for CMD, however, we asked women “Compared with other people of my same age and sex, I am less likely than they are to develop T2DM/hypertension or CVD.” Approximately 85% of women disagreed with this statement, meaning that the majority of participants were aware that they had the same or a *higher* risk for developing CMD compared with others (*i.e.*, low optimistic bias). The study conducted by Mukerji et al.^23^ suggested that assessing risk perception within the next 10 years may not be sufficient to accurately measure risk perception, which underscores the importance of assessing lifetime risk for future disease as well. In a follow-up question, women were asked “Compared with other people of my same age and sex, I am less likely than they are to develop a serious disease,” and only approximately 50% of the women agreed with this statement. This suggests that women underestimate the severity of CMD, which is very problematic, as it can lead to serious complications and be fatal. Targeted initiatives are needed to educate women about the long-term risks of APOs and their severity.

### Relationship between lifestyle modifications and risk perception for CMD

Overall, risk perception increased when women were asked to assess their risk perception of CMD if their current lifestyle did not change. Among women with PE, risk perception for hypertension and CVD increased from 43.5% to 63.0%, and among women with GDM, risk perception for T2DM increased from 45.6% to 63.0%. This increase in risk perception is encouraging, as it indicates that women recognize the significance of lifestyle modifications in the prevention of CMD. This is further supported by the fact that in our study, almost all women recognized a nutritious diet, regular exercise, and weight management as protective measures against the development of CMD. Furthermore, most women agreed that their personal efforts could mitigate their future risk of developing CMD. However, if women consider themselves to be at low risk for CMD, they may not see the value of or be motivated to adopt preventative measures, even if they understand the benefits of these measures. Therefore, increasing risk perception for CMD is vital; if women have a high risk perception for future CMD, they will have more motivation to enact change to decrease these risks, as high risk perception for a future disease is associated with increased health-seeking behaviors.^22,25–28^ This is reflected in our study: women with a high risk perception of CMD were three times more likely to plan to change their behavior to reduce their future disease risk. An Australian study^36^ assessed the knowledge and health-seeking behaviors of 438 individuals with PE or gestational hypertension. Although 62.2% of women were unaware of their increased risk of CVD, those who were aware were more likely to have regular CVD risk assessments and take antihypertensive medications. This further emphasizes the vital role of improving patient education to reduce the burden of CMD.

### Limitations

This study had several limitations. All women included in our study were recruited from a single hospital, making it challenging to apply the results to the general population. Furthermore, our sample sizes were unequal, with 115 women with GDM and 46 with PE. Unequal sample sizes lead to reduced statistical power, meaning that our statistical tests may have had a lower ability to detect significant differences or associations between groups or variables if they exist. Our survey was administered in German, excluding non-German speakers. Another limitation of our study was the selection bias. Since the telephone interview lasted approximately 30 minutes, women with an increased risk perception for future CMD may have been more interested in participating and learning than women with a lower risk perception. Regarding risk perception, women were only asked about their 10-year CMD risk; women may have had a higher risk perception if a longer time frame was used. Furthermore, we created a tool to ascertain risk perception among women with PE. This was not an established, validated tool such as the one used for GDM.

## Conclusion

This study provides crucial insights into the risk perceptions and behaviors of women with APO regarding their risk of future CMD. Women with GDM and PE had a low perceived risk of future CMD, especially notable in the PE group. However, when assuming that their lifestyles would remain unchanged, risk perception overall increased, suggesting an understanding of the importance of lifestyle changes. However, knowledge of this alone is insufficient. Our study found that women with a high perceived risk were three times more likely to plan to change their lifestyle, highlighting the critical role that risk perception plays in motivating lifestyle changes to mitigate long-term health risks.

This study underscores the urgent need for enhanced patient education tailored to women during pregnancy and the postpartum period. The HCP can play a crucial role in increasing risk awareness and motivating women to adopt healthier lifestyles. HCP need to be well informed about current guidelines and postpartum recommendations to effectively communicate risks and interventions to women with APO, highlighting the need for improved HCP training and knowledge. This approach can significantly reduce the burden of CMD among women diagnosed with APO, thereby improving long-term health outcomes (Figure 1).

## Data Access Statement

All data and materials are available from the corresponding author (B.P.) upon reasonable request.

## Abbreviations Used

APO: adverse pregnancy outcomes
BMI: body mass index
CMD: cardiometabolic disease
CVD: cardiovascular disease
GDM: gestational diabetes mellitus
HCP: health care providers
ICU: intensive care unit
PE: preeclampsia
SD: standard deviation
T1DM: type 1 diabetes mellitus
T2DM: type 2 diabetes mellitus
